# Biomechanics and neural control of movement, 20 years later: what have we learned and what has changed?

**DOI:** 10.1186/s12984-017-0298-y

**Published:** 2017-09-11

**Authors:** Andrew D. Nordin, William Z. Rymer, Andrew A. Biewener, Andrew B. Schwartz, Daofen Chen, Fay B. Horak

**Affiliations:** 1 University of Florida, PO Box 116131, Gainesville, FL 32611 USA; 2 Rehabilitation Institute of Chicago, Chicago, USA; 3 Northwestern University, Evanston, USA; 4 Harvard University, Cambridge, USA; 5 University of Pittsburgh, Pittsburgh, USA; 6 National Institute of Neurological Disorders and Stroke, Bethesda, USA; 7 Oregon Health and Science University, Portland, USA; 8 Veterans Affairs Portland Health Care System, Portland, USA

**Keywords:** Biomechanics, Motor control, Locomotion, Cortex, Spinal cord, BANCOM

## Abstract

We summarize content from the opening thematic session of the 20th anniversary meeting for Biomechanics and Neural Control of Movement (BANCOM). Scientific discoveries from the past 20 years of research are covered, highlighting the impacts of rapid technological, computational, and financial growth on motor control research. We discuss spinal-level communication mechanisms, relationships between muscle structure and function, and direct cortical movement representations that can be decoded in the control of neuroprostheses. In addition to summarizing the rich scientific ideas shared during the session, we reflect on research infrastructure and capacity that contributed to progress in the field, and outline unresolved issues and remaining open questions.

## Background

At the 20th anniversary meeting for Biomechanics and Neural Control of Movement (BANCOM), the opening thematic session was chaired by Dr. Fay Horak (Oregon Health & Science University). Presentations and discussions covered insights from 20 years of research in the field of motor control, delivered by Drs. Zev Rymer (Rehabilitation Institute of Chicago), Andy Biewener (Harvard University), Andy Schwartz (University of Pittsburgh), and Daofen Chen (National Institute of Neurological Disorders and Stroke). Presentation themes included the impact of technological advancements on motor control research, unresolved issues in muscle biology and neurophysiology, and changes in the scientific funding landscape. This brief review summarizes content presented by each speaker, along with discussions from the audience.

Considerable changes have occurred in the fields of biomechanics and motor control over the past 20 years, changes made possible by rapid technological advances in computing power and memory along with reduced physical size of biotechnology hardware. Because of these changes, research approaches have been reshaped and new questions have emerged. Previously, motor control research was constrained to laboratory-based assessments of individual neurons, muscles or joints, captured from low sample sizes. In the past, reliance on large, expensive, external recording devices, such as optical motion capture systems, understandably limited the feasibility of large-scale, multivariate research. Today, whole-body kinematic recordings using body-worn inertial measurement units, wireless electromyography (EMG), electroencephalography (EEG), and functional near infrared spectroscopy (fNIRS) systems, and electrode arrays for neural network recordings are increasingly commonplace. Alongside these technical leaps, sociocultural bounds have expanded research inclusion, as evidenced in the representation of speakers at the 2016 BANCOM meeting. In contrast to the 1996 meeting, which included three invited female speakers, 13 women were included as speakers in 2016. Such advancements will continue to shape our scientific landscape, driving innovation through new technologies and perspectives.

## Neuromuscular control: unfinished business

Although considerable progress has been made in the field of biomechanics and motor control over the past 20 years, there remains unfinished business on many fronts. Many tasks were halted because of technical obstacles that led to redirected research questions, though in some instances, loose ends have remained due to perceptions that remaining problems have already been solved. For example, a notable open question remains: “What do muscle spindle receptors sense?” We know that muscle spindles regulate muscle contraction by responding to changes in muscle length via changes in joint angle. However, the elaborate nature and more complex sensory function of these organs cannot be overstated. Matthews and Stein [1] revealed velocity-sensitivity of spindle afferents in detecting muscle length changes, but also identified non-linearities across stretch amplitudes. In response, Houk, Rymer, and Crago [2] tested the dynamic responsiveness of muscle spindle receptors during large stretches, revealing surprisingly weak velocity sensitivity. Instead, discharge rates were dependent on low fractional power of muscle lengthening velocity [2]. Houk and colleagues [2] also described friction-like features in the nonlinear dynamic response of loaded muscle spindles. The authors speculated that muscle control while moving inertial loads might be simplified by novel frictional damping, without the need for adjusting feedback gain [2]. Further research examining the nature of muscle length-velocity coding by muscle spindles is still warranted.

Overlap in the anatomical locations of alpha and gamma motoneurons in the ventral horn of the spinal cord is well-established [3, 4]. Their complex interrelationship, however, is less clear. Distinguishing gamma from alpha motoneurons can be accomplished using conduction velocity [3, 4], with gamma motoneuron axons conducting more slowly than alpha [4], resulting in a strict alpha-gamma coactivation order [5] during voluntary contractions. Gamma motoneurons lack group Ia monosynaptic connections such as those received by alpha motoneurons from homonymous muscles [4], but the reason for this difference remains unclear. Much of the work aimed at uncovering responses of human muscle spindles during muscular contractions has relied on tungsten microelectrodes inserted through the skin into relevant nerves. An arduous and low-yielding technique [6]. Such work is less common today, but is still worthwhile. Experimental paradigms examining muscle spindle afferent responses under isometric tension demonstrate a fixed, alpha-gamma coactivation strategy [5], though considerable variability exists in the recruitment thresholds among motoneurons [7]. Less orderly recruitment has also been observed in animal versus human muscle contractions [7, 8], exposing a necessity for further studies.

A variety of physiological motor control systems exist that appear to have emerged under evolutionary pressures [9]. Nerve fibers serve as communication channels, propagating action potentials through encoded messages transmitted along axons, which are encoded at peripheral receptors. The organization of communication channels and the subsystems to which they transmit messages therefore define control processes, including negative feedback, feedforward, and adaptive control [9]. Feedback control compares desired and actual performance in a closed loop. Feedforward control, however, is open loop, specifying goals a priori. Adaptive control modifies elements within a control system instead of directly and immediately altering output, thus working as a form of long-range planning. In both feedback and adaptive control, errors are evaluated from sensor signals as a form of model reference control [9]. Muscle spindles respond to changes in load by altering firing rate, thus adjusting controller properties [9]. Because motor commands are sent to both alpha and gamma motor neurons, co-contraction occurs between extra and intrafusal fibers. Anticipatory gamma motoneuron activation can, however, bias the muscle spindle’s stretch response as a form of feedforward control without the need for higher-level feedback, unless warranted by large errors [10].

The manner in which reference errors from muscle spindles are used to make adaptive adjustments at the level of the brain nevertheless remains incompletely understood. Muscle spindle discharge rates routinely increase during muscle lengthening, but also during shortening, especially when shortening is slow and against an inertial load. In each case, increased afferent activity has been attributed to the detection of movement irregularities that allow reflexive corrections [11].

Further insight into the role of muscle spindles in detecting joint movements has come from studies examining perceived movements during muscle vibration [6, 12–15]. Vibration-induced afferent signals have shown that increased muscle vibration frequency leads to increased discharge rate, up to a given threshold [6, 12], with perceived increases in lengthening velocity indicating the direction of movement [12]. This work has validated the function of muscle spindle afferents estimating muscle length and velocity [2], though detection of limb position appears to be dependent on approximations following vibration-induced illusory movements [12, 15]. Open questions remain on the relationship between muscle vibration and movement perception in biarticular muscles.

Understanding functional relationships between muscle spindle afferents and central motor control has consequences for neurological disorders. Spasticity is a known syndrome of stroke, multiple sclerosis, spinal cord injury, and central nervous system lesions, characterized by slow voluntary movements, exaggerated tendon reflexes, and muscle hypertonia expressed by rigid muscles with velocity-dependent stretch resistance [16]. Muscle hypertonia, a primary feature of spasticity has been attributed to overactive descending commands that lead to muscle over-activity from disinhibition [17], though the pathophysiology of muscle spasticity is, in fact, multifactorial [16]. Particularly puzzling is the lack of increased fusimotor drive in spastic muscles evidenced from microneurographic work [16], although these data were derived from passive muscles, which may not exhibit reflex behavior during voluntary motion. There is, therefore, a need for high fidelity intraneural recordings in humans with spasticity against matched controls, which might improve our understanding of interactions among descending commands, spinal level mechanisms, and modifications of skeletal muscle that contribute to spasticity following nervous system lesions.

Uncovering relationships between nervous system structure and function remains a considerable basic science challenge. Among the progress made in understanding the neural control of movement over the past 20 years is the dissection of spinal networks that control locomotion. Most notably, locomotor networks have revealed a distinct modular organization, where output from numerous integrated supraspinal areas interact with neuron assemblies located in the spinal cord to produce locomotor patterns and rhythms [18]. Despite the complexity associated with decoding neural signals, spinal circuits reveal an amalgam of afferent connections that appear to simplify locomotor function. Connections between left and right sides of the body, and between joint flexors and extensors enable rhythmic alternation during locomotion [18, 19]. Bilateral communication is made possible by commissural neurons with axons that cross the midline of the body, located in the ventral part of the spinal cord [18, 19]. Commissural neurons act on motor neurons, or interneurons, to provide inhibition for alternating muscle activation, or excitation for synchronous muscle activation. To control rhythmic flexion-extension muscle activation patterns, reciprocal Ia inhibition of motor neurons via upstream interneurons inhibits antagonist motor neurons [18, 20]. Stretch-activated group Ia afferents from muscle spindles enable reciprocal push-pull using alternating circuits. In each case, functional modularity underlies the basic structure of the nervous system at the level of the spinal cord. This fundamental understanding of the complexity of spinal control of locomotion had profound implications for technological solutions designed to activate spinal locomotor circuitry in spinal cord injured humans.

Detailing interactions between upstream motor commands and spinal locomotor networks remains unresolved. Previously, motor maps charted functional relationships between motor cortical areas and muscles associated with specific joint movements. Although more complex than simple topographic cortical representations of muscle activations, the motor cortex depicts limb movements [21]. Contemporary research combining a variety of techniques, including gene expression and transcription [19, 20], as well as measurements from vast neuronal arrays, has the ability to unveil new information about the manner in which cell populations interact and produce behavior [18]. Connections between nervous system organization and functional connectivity patterns will therefore continue to advance our understanding of movement control.

## Complex motor tasks and locomotor control

Among the challenges faced in understanding motor control mechanisms is the need for in vivo muscle function examinations during complex motor tasks. To this end, we summarize recent findings across muscles and species, including interactions among muscle properties and neural reflexes during motor learning and task execution, with an emphasis on locomotion.

Studies examining in vivo muscle function have revealed time-varying force-velocity and force-length relationships. Neural recruitment interacts with muscle activation patterns to regulate muscle force, length, and work, which affect the cost of force generation. Tendon stretch has been outlined as a key element for reducing locomotor energy cost, therefore muscle architecture and physiology combine with tendon geometry for specific functions [22]. Changes in muscle-tendon forces have been measured using embedded force transducers [23] and when combined with muscle fascicle length measurements from sonomicrometery, can be used to record dynamic force-length behaviors that allow mechanical work and power calculations [22]. In combination with EMG measures, relationships between muscle activation and force-length changes provide insight into neuromuscular control [22]. Using these approaches, dynamic muscle responses during locomotion have been studied.

Under concentric conditions, muscles shorten to produce mechanical work, maximizing power in movements such as climbing and jumping. During hopping and running, muscles may contract nearly isometrically, undergoing small length changes and performing little work while producing large forces. Reduced energy cost is therefore achieved by recovering elastic energy from tendon stretch, as well as by reducing the recruited volume of muscle needed to generate force [22]. In contrast, energy absorption is achieved during eccentric contractions by active muscle lengthening, such as during landing, which often occurs with a brief stretch prior to muscle shortening. Relationships between muscle-tendon architecture and function have therefore been revealed. Short muscle fascicles with pennate architecture are well suited to produce forces economically, with long thin tendons providing elastic energy savings. Alternatively, long muscle fascicles with absent or little tendon enhance mechanical power and position control [22].

Biomechanical observations during locomotion have uncovered much about the basic principles of locomotor control. In vivo observations have revealed that neuromechanical interactions among intrinsic muscle contractile properties, reflex feedback and feed-forward control are key to stabilizing rapid movements. Comparative research has therefore been carried out across species, demonstrating fundamental underlying similarities. Many studies have relied on the simple spring-mass model for explaining dynamic energy fluctuations of the body’s center of mass, based on a ‘spring-loaded inverted pendulum’ [24, 25]. Because isometric, eccentric, or concentric muscle contractions rarely occur in isolation during normal locomotion, in vivo force and muscle activation measurements have exposed elastic energy recovery mechanisms and force sharing strategies in the plantarflexors of freely moving cats [26–28]. Tendon stretch and recoil in running turkeys [29] and guinea fowl [30, 31], as well as hopping wallabies [32], have further shown energy savings using nearly isometric plantarflexor force development. However, greater muscle shortening has been shown to increase work output during incline locomotion in turkeys [29], guinea fowl [30], and goats [33]. The ability of distal limb muscles to generate muscle forces isometrically or with low shortening velocities, reduces mechanical work and subsequent metabolic energy cost [30]. While running over complex terrain, guinea fowl achieve stabilization from the intrinsic properties of distal muscle-tendon structures in combination with limb and body dynamics, described by the spring-mass model [31, 34]. Distal muscles therefore appear to transmit forces using elastic energy storage in tendons, with proximal muscles showing more complex recruitment and in vivo length change patterns, seemingly under feedforward control, to produce spring-like limb behavior [31, 34].

Locomotion speed also plays a role in the ability of higher brain centers to maintain stability. At fast speeds, spinal sensorimotor circuits must work in concert with the biomechanical properties of the musculoskeletal system to maintain stability due to transmission delays from higher level neural control centers [34].

Although overground locomotion has been studied in a number of species, comparative muscle function has also been evaluated in bird flight, which further underscores relationships between muscle-tendon design and function. During flight, the pectoralis muscle of the pigeon undergoes isometric or eccentric strain in late upstroke, enhancing force development, before shortening over substantial length during downstroke and performing work [35]. The long parallel fascicles of the avian pectoralis muscle share architectural features similar to proximal limb muscles in running animals, but are in contrast with the short gastrocnemius fascicles in cats, wallabies and turkeys, and the highly pennate muscle fibers of ungulates [22, 35]. In a comparative context, an inverse relationship appears to exist between high strain, low-energy economy, parallel-fibered muscles and low strain, high-energy economy, pennate-fibered muscles that recover elastic energy from longer tendons. Muscle architecture is therefore fundamentally linked to neural recruitment patterns to provide effective and robust motor control strategies.

Obstacle avoidance strategies in running guinea fowl indeed suggest that locomotor stability when moving across uneven terrain is maintained using a combination of feedforward adjustments and reflex feedback to tune leg impedance [36]. Because direct in vivo proximal muscle work calculations are not possible, EMG recruitment patterns combined with inverse dynamics analysis of ground reaction forces and movement kinematics must be relied on instead [34]. These approaches can be validated against neuromechanical simulations to study possible locomotor control mechanisms of the musculoskeletal system, including in humans. Further studies using simulations and when moving over natural terrains are therefore needed.

With the growing use of musculoskeletal modeling and neuromuscular simulation to study normal and pathological gait, there is a need for improved muscle models. Wakeling [37] demonstrated time-varying shifts in motor unit recruitment patterns during human walking and running, indicating that different motor unit types are recruited in a task-dependent manner. Because whole muscles are typically heterogeneous with respect to fiber type, containing a mixture of slow and fast contractile fibers, Hill-type muscle models incorporating single contractile element types likely limit the accuracy of musculoskeletal modeling predictions [37, 38]. For this reason, differential muscle models that include fast and slow motor units have been developed and shown to improve muscle force predictions [38]. This work has incorporated time-frequency wavelet analysis that characterizes differences in the frequency content of EMG signals over time, encoded by motor units with contrasting contractile properties [38]. Faster motor units have greater conduction velocities than slower motor units, leading to greater spectral content at higher EMG frequencies and time-varying force development rates not captured in traditional muscle models [38]. Improved muscle force predictions using differential Hill-type muscle models that incorporate fast and slow motor unit properties, together with improved knowledge of passive and active muscle force-length relationships, can therefore better represent in vivo locomotor performance. Accounting for the inertial effects of internal muscle mass will likely further improve simulated contractile performance [39]. Although the inertial effects of tissue mass are largely ignored in Hill-type muscle models, delays in the time to reach maximum shortening velocity have recently been shown in submaximal activations. Under these conditions, contracting muscle fibers must work to accelerate their own mass along with the mass of inactive fibers [39].

Beyond the need for improved muscle models for calculating in vivo muscle loads, examining the role of cortical circuits in motor skill learning and task execution is critical for understanding more complex features of the neural control of movement. Neuroanatomical approaches that relate isolated brain regions to specific behaviors assume direct relationships between structure and function. Increasingly, however, research examining transient perturbations of specific neural circuits uncovers interconnections within the brain that can compensate to recover learned motor tasks [40]. The ability of the brain to recover from lesions therefore appears to be driven by repurposing unaffected circuits [40]. Following permanent lesions to rat motor cortex, pre-lesion behaviors were recovered, suggesting that homeostatic regulation of neuronal dynamics might enable functional recovery after brain injury [40]. In these experiments, rats learned a precise spatiotemporal sequence of lever presses that provided a liquid reward [40, 41]. After lesioning the motor cortex, the animals were initially impaired on the contralateral side, indicative of subcortical motor circuits having lost cortical inputs. After 10 days of recovery, however, basic motor functions had been restored, and after 11 days the animals were able to perform the previously learned motor sequence [41]. However, when the motor cortex of untrained rats was lesioned, rats were unable to acquire the sequence, suggesting the motor cortex tutors subcortical motor circuits during learning [41]. Motor sequence acquisition and consolidation can therefore be reprogrammed, but only if previously learned, with the motor cortex overseeing downstream motor output while incorporating contextual and sensory information useful for planning [41].

Behavioral studies examining motor variability in humans and animals provide another window into motor control processes. Motor variability has been used to measure motor learning, revealing higher levels of task-relevant variability in early skill acquisition [42]. Trial-to-trial variation is nevertheless inevitable during motor repetition. Typically, motor control processes are thought to minimize variability in a signal dependent manner, scaled to the size of the motor output [42]. Emerging evidence, however, suggests the nervous system not only regulates motor variability, but in fact amplifies variation during learning [40]. In humans, greater task-relevant variability while learning point-to-point arm reaching trajectories, and while experiencing a velocity-dependent force field, were shown to predict faster learning [42]. Performance accuracy might therefore be sacrificed in favor of motor exploration during learning [42]. Although motor variation is reduced when precision is crucial, the motor system appears to restructure temporal features of motor variability to enhance learning. Bridging connections between movement mechanics and activation patterns of cortico-spinal neural circuits is therefore necessary to understand motor learning and improve treatment of motor deficits.

## Cortical movement control and neuroprostheses

The manner in which the brain controls movement has remained a central basic science question over the past 20 years of motor control research. Technological and computational advancements over the past decades have enabled many new discoveries in this area of research. Circa the 1996 BANCOM meeting, single unit recordings from the motor cortex of monkeys allowed hundreds of action potentials to be measured extracellularly. When analyzed as a population, relationships between cortical firing rates and movement velocity were uncovered, demonstrating that movement velocity was continuously encoded in motor cortex and could be extracted as a spatiotemporal trajectory [43–45]. Continual research has extended these findings to humans and has revolutionized brain-machine interfaces for prosthetic control. We devote this section to the current knowledge of cortical movement control and remaining research challenges.

The last 20 years of research into the cortical control of arm and hand movement has emphasized the idea of population coding. Rather than each neuron coding for one particular parameter (e.g. contraction of a specific muscle), it has become clear that each neuron encodes multiple movement parameters simultaneously. If all of these parameters are together encoded as a single firing rate, predicting all of the parameter values from an individual neuron would not be possible. Because these parameters may be weakly but redundantly represented in these individual neurons, a de-multiplexing or decoding algorithm, using a population of the firing rates recorded from many individual neural units can be used to extract consistent predictions of their values. In a series of experiments [46–51], Georgopoulos and colleagues showed neural evidence for cortical representations of arm movements in primates by computing the neuronal population vector from cellular activities in the motor cortex across successive time intervals. During a center-out task involving arm movements from a center location to one of eight equally spaced targets, neuronal discharge rates in the motor cortex were plotted against movement direction [46]. When fit with a cosine function, discharge rates were observably modulated by movement direction. As a result, although individual neurons show directional preference, when considered as components of a vector, each cell makes a weighted contribution along its preferred direction. Arranging the vectors tip to tail in the form of a time series therefore depicts the intended movement trajectory, collectively known as the population vector algorithm [47–51]. From these experiments, neuronal populations were found to accurately represent arm velocity. Movement direction and speed are continuously represented by motor cortical discharge rates as a true velocity vector while moving the hand along a specified trajectory. During drawing movements in monkeys, Schwartz showed that population vector length and direction varies with the movement trajectory, preceding movement by approximately 120 ms [52]. Importantly, when moving one’s arm along a curved path, speed is inversely proportional to the curvature of its path [53]. The population vector produced from motor cortical discharge rates should therefore follow the same relation. Schwartz indeed confirmed the so-called “2/3 power law” in the motor cortical activity of monkeys, though important temporal features emerged in spiral drawing [53]. Here, the time lag between the neural population vector and the observed hand movement increased as the curve became tighter [45, 53]. Similar findings during lemniscate, or figure eight, drawing suggest that predictive movement representations in the motor cortex further precede movement as the curvature radius decreases [43]. Segmentation of motor planning and execution therefore appears to be an important feature of drawing movements [43]. This work emphasized the kinematic parameters of movement with less consideration of the muscle activations used to propel the arm and hand [43–45]. The applications of these findings are already apparent in the cortical control of neuroprostheses for individuals who are paralyzed and unable to use their muscles.

Extracting the neural representations of movement has been made possible by multichannel microelectrode arrays implanted in the motor cortex of primates, and, more recently, humans. Commonly-used microelectrode arrays that enable long-term recordings include the planar Michigan silicon probe with multiple recording sites per probe [54] and the three-dimensional Utah array [55, 56]. The Michigan probe has been used to control neuroprostheses in monkeys from 60 to 80 individual units [57], while the Utah array provides 100 separate channels per array [55, 56]. When combined with real-time decoding algorithms, including a modified population vector algorithm [58] or Kalman filter [59, 60], implanted microelectrode arrays have been used to control three-dimensional robotic arm movements from neuronal population signals across multiple cortical areas [58, 61]. In monkeys, signals recorded from intracortical microelectrode arrays have been used to control a seven degree-of-freedom robotic arm for self-feeding [62], though continual progress in neural prosthetics have extended this control to more dimensions. Nowhere is this more apparent than in the concerted effort by the Defense Advanced Research Projects Agency (DARPA) Revolutionary Prosthetics program, which has developed state-of-the-art neuroprostheses. The Johns Hopkins University/Applied Physics Lab Modular Prosthetic Limb offers 17 degrees of freedom in 26 articulating joints with extensive dexterous capabilities provided by fingers and a thumb [63]. In humans, neuroprostheses have direct applications for individuals with movement disorders and paralysis. For this reason, neuronal ensembles recorded from an individual with tetraplegia were used to perform virtual computer tasks and to control a simple multi-jointed robotic limb [64]. Not only did this study demonstrate that microelectrodes implanted into the human primary motor cortex could control a neuroprosthesis, but also that cortical spiking patterns persisted 13 years after spinal cord injury [64]. Because robust neuroprosthesis control has relied on intracortical recordings from implanted microelectrode arrays, efforts to extract signals from the cortical surface (electrocorticography, ECOG) [63, 65], or from the scalp using non-invasive EEG [66, 67], continue to advance brain computer interfaces. Some rudimentary hand and finger control has been demonstrated using signals recorded on the cortical surface of humans during slow, unconstrained grasping movements [65], and intracortical microstimulation has been used to provide somatosensory feedback in monkeys [68].

More recent work has shown how the high-performance Modular Prosthetic Limb can be controlled as a neural prosthetic device. A paralyzed woman implanted with intracortical microelectrode arrays in her motor cortex was able to control this device using 10 degrees-of-freedom. She was able to control 3D arm displacement, 3D wrist orientation, and four dimensions of hand shape *simultaneously* to produce graceful, coordinated reach, grasp, and placement of objects in tasks exemplifying those that occur in acts of daily living [69]. Using the same paradigm based on intracortical microelectrodes in the motor cortex, another subject implanted with additional arrays in his sensory cortex was able to perceive tactile activation of the prosthetic fingers through intracortical microstimulation of these additional electrodes [70].

Advancements in the area of neuroprostheses will continue to occur on several fronts. Robotic limbs that supply full manual dexterity and somatosensory feedback are in development, along with advanced neural decoding algorithms applied in real time. The ability to control the compliance or impedance of the robot effector is an active area of research and its development is essential for dexterous object manipulation. Intracortical microelectrode arrays have shown to be capable of recording signals from which high-fidelity, many degree-of-freedom arm and hand control signals can be robustly extracted. The information contained in these signals is much greater than that found in the mixed neuron recordings provided by ECOG or EEG. Intracortical recordings are often faulted for the limited duration (months to years) in which reliable single-units can be isolated from the recordings. However, even though the number of isolatable single units may decrease, multi-unit recordings persist almost indefinitely. High-fidelity control signals can also be extracted from these multi-unit recordings [71]. In addition, a number of technical advances in electrode design are increasing tissue-electrode compatibility with the promise of long-term chronic single-unit recordings [72–74]. The development of wireless technology to telemeter the recorded signals, promises to eliminate the need for the skull-mounted transcutaneous connectors used currently [75], which will reduce the risk of possible infection and damage to the connector.

Although further work is needed to enable real world functionality for neuroprostheses, considerable progress has been achieved by harnessing the results from years of basic research describing the relation between cortical activity and movement. This fundamental knowledge has forged new paths for applied research, underscoring the importance of basic science research in generating bottom up growth.

## Building and sustaining research capacity

Advances in computing power and micro-electromechanical systems have indeed been a key driving force behind the growth of biomechanics and neural control of movement as a significant field. Increases in both human and financial resources, however, have also grown in recent decades, which have expanded research capacity. The emergence and maturation of biomedical engineering as an academic discipline has not only provided additional intellectual drive and analytical approaches to tackle fundamental movement control research questions, but has also provided a highly skilled research workforce that is capable of taking full advantage of newly available tools. At the same time, a desire for achieving clinical autonomy and evidence-based practices further enabled the emergence of clinical rehabilitation as an applied research area. The two forces formed an unprecedented synergy, and created new opportunities for clinical research. In North America, early research clusters of critical mass played a critical role for incubating and nurturing a nascent but growing research capacity in biomechanics and neural control of movement. Most critically, in a 5-years period following the 1996 BANCOM meeting (1998-2003), the *National Institutes of Health* (NIH) saw its budget doubled. This significant funding increase was timely for mushrooming biomedical engineering departments in major research institutions across the US, launching many biomechanics, motor control, and rehabilitation research focused graduate programs. Established funding programs relevant to the field of biomechanics and neural control of movement at the NIH dramatically increased the number of funded applications, including those at the *National Institute of Neurological Disorders and Stroke* (NINDS), the *National Institute on Aging* (NIA), and the *National Institute of Arthritis and Musculoskeletal and Skin Diseases* (NIAMS). Furthermore, the newly legislated and formed *National Center for Medical Rehabilitation Research* (NCMRR) and the *National Institute of Biomedical Imaging and Biomedical Engineering* (NIBIB) made additional resources available, and were active in joining the growing critical mass of NIH funding programs focused on developing scientific initiatives with various funding mechanisms essential in building research capacity in the area of biomechanics and neural control of movement. Of all the NIH programs that promoted research funding in this area, the NINDS Neuroprosthesis Program is widely considered to have played a critical role in effectively boosting support for cutting-edge and risk-taking innovative research. Importantly, many efforts were made through special research contract mechanisms, which proved to be effective for addressing challenges associated with an initial lack of expert representation in the competitive NIH peer-review process of unsolicited proposals, particularly in the neural interface field that was still in its adolescence. Other special funding programs that played pivotal and sustained roles for the growth of this field include the NIH-NSF (National Science Foundation) joint *Collaborative Research in Computational Neuroscience* (CRCNS) and the *Bioengineering Research Partnership/Grant programs* (BRP/BRG).

In addition to the NIH, several other mission-specific federal funding programs, including those at the *US Department of Veterans Affairs* (VA), the *National Science Foundation* (NSF), and the *National Institute on Disability, Independent Living and Rehabilitation Research* (NIDILRR), all played significant roles in facilitating the development of early research clusters, mostly through their training and center grants. Special funding programs aimed at addressing the need for protection and care of warfighters at the *US Department of Defense* (DoD) and DARPA accelerated the pace for innovative translational efforts aimed at restoring, maintaining, and enhancing mobility, as well as other biomechanical and sensorimotor capabilities of warfighters using biologically inspired systems for defense technologies. This surge in research capacity has been transformative for the field, reflected not only in the increasing number of funded investigators, but also in topic diversity generated from multidisciplinary collaborations. As a result of a 3-4 fold surge in the number of NIH applications in the area of biomechanics and neural control of movement in the years following the NIH budget doubling, the NIH Center for Scientific Review (CSR) expanded review panels to accommodate new peer-review demands. These additional review needs were implemented first through ad hoc special emphasis panels (SEPs) and then through specially chartered and themed study sections. Most notably, the *Motor Function and Speech Research* (MFSR) and the *Musculoskeletal Rehabilitation Sciences* (MRS) study sections evolved during that time, in addition to the already existing *Sensorimotor Integration* (SMI) panel, which together conducted reviews for significant portion of the investigator-initiated proposals submitted to NIH. Currently, the SMI, MFSR, and MRS remain the most significant study sections concentrated on proposals themes related to biomechanics and movement control, with an increasing proportion of applications in this area also being reviewed by other panels with expertise in cognitive, disease mechanisms, or technological development components. Serving now as the formalized review channel for applications in this program area, these CSR panels not only reflect robust growth, but also symbolize an emerged *identity* for a branch of science that was ready in both breadth and depth to compete with other scientific areas for research support from a common NIH resource pool for investigator-initiated proposals. This increase has been steady over the years, even after the NIH budget stalled and then flattened since the mid-2000s. The growth of this multidisciplinary domain coupled with a shared desire to understand neural control of human movement and a shared interest in addressing clinically relevant movement problems was well represented by many of the young BANCOM attendees in 1996. These investigators later became part of the significant cohort of applicants to take advantage of the NIH’s well-established peer-reviewed system with well defined and consistent review criteria [76], competing successfully with their investigator-initiated proposals.

From the perspective of sustainability for any scientific area, having a critical mass of NIH R01 funded investigators is essential, as the funding mechanism is considered to be the only *renewable* research project grant of its kind for *unsolicited* proposals across the entire research funding ecosystem.

Moving forward, it is essential to maintain a broad and balanced portfolio of basic, translational, population, and clinical research for achieving expected outcomes and public health benefits. Because uncovering the structure and function of the brain remains a substantial basic science challenge, it is particularly critical to continue vigorous support in our pursuit of fundamental knowledge [77]. The NIH has repeatedly emphasized its continuing commitment to basic biological science, especially after a recent portfolio analysis indicating a “gradual and significant decline in the number of basic grants awarded between 1997 and 2012” [78]. This decline apparently was found to be due to a decrease in submissions of basic research applications to NIH, even when the basic grant applications did better in peer review than applied research proposals [79]. In response, NIH institutes such as NINDS have set aside funds specifically to boost basic science research efforts under a “back to basics” initiative, while also adopting policies to enhance research quality by emphasizing scientific premise, rigor, and reproducibility [80].

Like many other scientific research areas, the field of biomechanics and neural control of movement must rely on an iterative process from basic, translational, to clinical studies, as well as reverse-translation for mechanistic insights using animal models [81]. Being inherently multidisciplinary and complex in nature, research progress in human movement science relies on sustained research capacity and synergized collective efforts by investigators from different levels or generations [82, 83]. Preserving this strategy will ensure that our intellectual heritage can be protected and further enriched, and that both old and new theoretical frameworks can be further evolved or developed.

Given the migration and evolution of research capacity over the past decades, an ideal academic home for the field of biomechanics and neural control of movement may vary from institution to institution, depending on overall institutional strength and research focus. Nevertheless, in-depth training in fundamental systems neuroscience and human physiology are essential elements for training programs studying human sensorimotor control, nurturing a regenerative process for basic science ideas, be they based in biomedical engineering, kinesiology, or rehabilitation science. In a time of budget constraints and evolving landscape for the academic research enterprise, it is crucial for current and future research capacity growth to be maintained or focused particularly on research addressing basic and fundamental questions, so that the entire field studying biomechanics and neural control of movement can continue to be robust and resilient [84, 85].

## Conclusions

In this review, we summarized many scientific discoveries from the past 20 years of research in the field of biomechanics and neural control of movement. Rapid technological, computational, and financial growth have led to the development of fundamental scientific knowledge in the context of communication mechanisms at the level of the spinal cord, relationships between muscle structure and function, and direct cortical representations of movement that can be decoded to control neuroprostheses. Despite the depth and breadth of these discoveries, unresolved questions remain, particularly surrounding the manner in which various levels of the nervous system interact to produce and control movement. The need to generate more testable hypotheses in uncovering the neural basis of movement control therefore becomes clear. Academic challenges in training the next generation of motor control scientists to include a firm background in basic neuroscience, as well as translational and clinical expertise, will hopefully be resolved over the next 20 years. Although an incomplete synopsis of two decades of biomechanics and neural control of movement research, we hope this review provides insight into the rich scientific ideas shared in the opening thematic session of BANCOM 2016.

## Abbreviations

BANCOM: Biomechanics and neural control of movement
BRP/BRG: Bioengineering Research Partnership/Grant programs
CRCNS: Collaborative Research in Computational Neuroscience
CSR: Center for Scientific Review
DARPA: Defense Advanced Research Projects Agency
DoD: US Department of Defense
ECOG: Electrocorticography
EEG: Electroencephalography
EMG: Electromyography
fNIRS: Functional near infrared spectroscopy
MFSR: Motor function and speech research
MRS: Musculoskeletal Rehabilitation Sciences
NCMRR: National Center for Medical Rehabilitation Research
NIA: National Institute on Aging
NIAMS: National Institute of Arthritis and Musculoskeletal and Skin Diseases
NIBIB: National Institute of Biomedical Imaging and Biomedical Engineering
NIDILRR: National Institute on Disability, Independent Living and Rehabilitation Research
NIH: National Institutes of Health
NINDS: National Institute of Neurological Disorders and Stroke
NSF: National Science Foundation
SEP: Special emphasis panel
SMI: Sensorimotor Integration
VA: Department of Veterans Affairs
